# Complications, Deaths, and Disability Burden in the 2 Years Following Dengue Infection

**DOI:** 10.1001/jamanetworkopen.2025.59108

**Published:** 2026-02-12

**Authors:** Jo Yi Chow, Wei Zhi Tan, Liang En Wee, Peihong Guo, Esther Li Wen Choo, Calvin Chiew, Lalitha Kurupatham, Lee Ching Ng, Po Ying Chia, David Lye, Kelvin Bryan Tan, Jue Tao Lim

**Affiliations:** 1 National Centre for Infectious Diseases, Singapore; 2 Lee Kong Chian School of Medicine, Nanyang Technological University, Singapore; 3 Duke-NUS Graduate Medical School, National University of Singapore, Singapore; 4 Department of Infectious Diseases, Singapore General Hospital, Singapore; 5 Communicable Diseases Agency, Singapore; 6 Ministry of Health, Singapore; 7 National Environment Agency, Singapore; 8 Department of Infectious Diseases, Tan Tock Seng Hospital, Singapore; 9 Yong Loo Lin School of Medicine, National University of Singapore, Singapore; 10 Saw Swee Hock School of Public Health, National University of Singapore, Singapore

## Abstract

This cohort study estimates the 2-year risks, rates, and disability-adjusted life-year burden of postacute, multisystem sequelae of dengue infection in Singapore, where dengue is endemic with recurrent outbreaks.

## Introduction

Prior studies^1,2^ of postacute dengue sequelae report its association with subsequent cardiovascular, neuropsychiatric, and gastrointestinal complications, but follow-up in those studies was short, and estimates of the disability-adjusted life-year (DALY) burden were limited. We estimated the 2-year risks, rates, and DALY burden of postacute, multisystemic sequelae following dengue infection in Singapore, where dengue is endemic with recurrent outbreaks.

## Methods

We conducted a retrospective cohort study using Singapore’s linked national dengue surveillance and health care claims data (eAppendix 1 and eFigure in Supplement 1). Institutional review board approval and patient consent were not required as this study was conducted under the Infectious Diseases Act, Singapore. Reporting followed the STROBE reporting guidelines for cohort studies.

Adults aged 18 years and older with dengue first notified as of time 0 (T_0_) between January 1, 2013, and June 30, 2022 were included. Individuals who died 30 days or less after T_0_, had another dengue infection 30 to 730 days after T_0_, or SARS-CoV-2 infection 365 days before T_0_ or 30 to 730 days after T_0_ were excluded. Controls had no recorded dengue and were alive 30 days after an assigned enrollment date sampled from the distribution of case enrollment times, yielding 42 controls per case by exact matching.

Primary outcomes from T_0_ plus 30 days to T_0_ plus 730 days were all-cause mortality, hospitalization, and incident cardiovascular, neuropsychiatric, autoimmune, kidney, endocrine, and gastrointestinal sequelae defined by *International Statistical Classification of Diseases and Related Health Problems, Tenth Revision *codes, recorded in national health care claims data (eAppendix 2 in Supplement 1). Covariates included age, sex, ethnicity (from national registration records), Charlson Comorbidity Index, prior admissions, and housing-based socioeconomic status. Propensity scores were estimated using logistic regression with overlap weighting, and standardized mean differences (SMDs) assessed covariate balance. Competing-risks regression (death as competing event) and Cox models were used to estimate hazard ratios (HRs); negative-binomial regression was used to estimate incidence rate ratios (IRRs) and excess burdens (weighted incidence rate differences). Excess DALYs were derived by multiplying excess burdens by Global Burden of Disease 2021 Level-3 DALYs per case. Subgroup analyses included age, sex, ethnicity, socioeconomic status, and comorbidity to examine potential effect modification. Sensitivity analyses applied alternative weighting strategies, DALY specifications, negative-outcome control (asthma), and temporal incidence assessments. All statistical tests reported 95% CIs, with statistical significance defined as a 95% CI that did not include 1. Analyses were implemented in R statistical version 4.3.1 (R Project for Statistical Computing) (eAppendix 3 in Supplement 1).

## Results

After exclusions, 68 145 dengue-infected adults and 2 886 119 controls were included. SMDs were below 0.1 after weighting (Table 1). Over 2 years, dengue infection was associated with increased risks of all-cause hospitalization (HR, 1.12; 95% CI, 1.10-1.14) and any prespecified sequelae (HR, 1.06; 95% CI, 1.01-1.11) (Table 2). We estimated 1670 excess DALYs (95% CI, 850-2492 excess DALYs) (Table 2) due to sequelae, equivalent to 2.52 DALYs per 100 infections. Neuropsychiatric sequelae contributed most of the burden (1589 DALYs), followed by endocrine (53 DALYs), kidney (16 DALYs), and gastrointestinal (4 DALYs) sequelae. Although risks for postacute sequelae declined across most organ systems in the second year, elevated risks for all-cause hospitalization and for neuropsychiatric, kidney, and gastrointestinal complications persisted. Excess DALYs were higher among older adults (aged ≥61 years), individuals with comorbidities, public housing residents, male individuals, and those of Chinese ethnicity.

**Table 1.** Baseline Characteristics Before and After Overlap Weighting

Characteristic	Unweighted		Weighted		SMD^a^
	Controls (n = 2 886 119)	Dengue positive (n = 68 145)	Controls	Dengue positive	
Age, mean (SD), y	45.87 (16.96)	44.6 (16.42)	44.62 (17.00)	44.62 (16.41)	<.01
Ethnicity					
Chinese	2 179 522 (75.52)	53 916 (79.12)	52 094 (78.99)	52 094 (78.99)	<.01
Indian	262 614 (9.10)	5536 (8.12)	5379 (8.16)	5379 (8.16)	<.01
Malay	339 319 (11.76)	6833 (10.03)	6663 (10.10)	6663 (10.10)	<.01
Others^b^	104 664 (3.63)	1860 (2.73)	1812 (2.75)	1812 (2.75)	<.01
Sex					
Female	1 505 634 (52.17)	31 588 (46.35)	30 670 (46.51)	30 670 (46.51)	<.01
Male	1 380 485 (47.83)	36 557 (53.65)	35 273 (53.49)	35 273 (53.49)	
Apartment type^c^					
1-2 Rooms public	134 812 (4.67)	2346 (3.44)	2296 (3.48)	2296 (3.48)	<.01
3 Rooms public	439 230 (15.22)	8286 (12.16)	8096 (12.28)	8096 (12.28)	<.01
4 Rooms public	915 902 (31.73)	17 949 (26.34)	17 538 (26.59)	17 538 (26.59)	<.01
5 Rooms or executive public	1 119 839 (38.80)	26 635 (39.09)	25 916 (39.30)	25 916 (39.30)	<.01
Others	66 511 (2.30)	468 (0.69)	463 (0.70)	463 (0.70)	<.01
Private	209 825 (7.27)	12 461 (18.29)	11 638 (17.65)	11 638 (17.65)	<.01
Charleson Comorbidity Index ≥1	299 715 (10.38)	7577 (11.12)	7345 (11.14)	7345 (11.14)	<.01
Previous hospitalizations (≥1)	975 746 (33.81)	35 994 (52.82)	34 455 (52.25)	34 455 (52.25)	<.01

Abbreviation: SMD, standardized mean difference.

^a^ Postmatching SMD of less than 0.05 denotes good balance in that specific characteristic between the controls and dengue-positive groups.

^b^ Includes individuals of other ethnicity (eg, Eurasian or Arab) or multiple ethnicities.

^c^ Apartment type was used as a proxy for socioeconomic status.

**Table 2.** Postacute Sequelae 2 Years After Dengue Infection Among Patients and Control Groups

Outcome	30-365 d After infection^a^			365-730 d After infection^a^		
	HR (95% CI)	IRR (95% CI)	Excess DALYs (95% CI)	HR (95% CI)	IRR (95% CI)	Excess DALYs (95% CI)
Death	1.93 (1.68 to 2.21)^b^	1.81 (0.69 to 4.76)	NE^c^	0.89 (0.77 to 1.03)	1.04 (0.25 to 4.41)	NE^c^
All-cause hospitalization	1.13 (1.11 to 1.15)^b^	1.18 (1.14 to 1.22)^b^	NE^c^	1.12 (1.10 to 1.14)^b^	1.14 (1.11 to 1.17)^b^	NE^c^
Composite outcomes						
Any sequelae	1.12 (1.07 to 1.17)^b^	1.18 (1.08 to 1.29)^b^	NE^c^	1.06 (1.01 to 1.11)^b^	1.03 (0.94 to 1.13)	NE^c^
Major adverse cardiovascular event	1.20 (1.05 to 1.37)^b^	1.17 (0.94 to 1.46)	22.64 (−3.40 to 48.68)	1.05 (0.91 to 1.21)	0.99 (0.79 to 1.24)	−1.72 (−26.19 to 22.74)
Autoimmune outcomes	1.22 (0.86 to 1.73)	1.02 (0.42 to 2.48)	2.07 (−20.64 to 24.79)	0.91 (0.63 to 1.33)	0.54 (0.24 to 1.18)	−35.16 (−55.79 to −14.53)
Cardiovascular and thrombotic outcomes	1.12 (1.00 to 1.25)	1.33 (0.54 to 3.31)	25.26 (−0.73 to 51.25)	1.05 (0.94 to 1.18)	0.99 (0.82 to 1.18)	−2.72 (−27.80 to 22.36)
Neurological and psychiatric outcomes	1.21 (1.09 to 1.33)^b^	1.30 (1.09 to 1.55)^b^	939.49 (499.68 to 1379.30)^b^	1.09 (0.99 to 1.21)	1.18 (0.99 to 1.40)	572.98 (129.89 to 1016.06)^b^
Endocrine outcomes	1.12 (1.06 to 1.18)^b^	1.21 (1.09 to 1.34)^b^	27.23 (19.40 to 35.060)^b^	1.07 (1.01 to 1.13)^b^	1.05 (0.95 to 1.16)	7.61 (−0.49 to 15.71)
Gastrointestinal outcomes	1.24 (1.04 to 1.47)^b^	1.21 (0.92 to 1.58)	1.29 (−0.41 to 2.99)	1.24 (1.05 to 1.47)^b^	1.38 (1.05 to 1.81)^b^	2.41 (0.60 to 4.22)^b^
Kidney outcomes	1.98 (1.46 to 2.68)^b^	2.14 (0.77 to 6.03)	7.84 (3.64 to 12.04)^b^	1.05 (0.72 to 1.54)	1.39 (0.59 to 3.28)	4.86 (0.69 to 9.02)^b^
Individual diagnoses						
Autoimmune						
Connective tissue disorders	1.84 (1.05 to 3.21)^b^	1.96 (0.54 to 7.17)	1.29 (0.14 to 2.44)^b^	0.82 (0.39 to 1.74)	0.60 (0.14 to 2.50)	−0.67 (−1.64 to 0.29)
Cardiovascular						
Dysrhythmias	1.23 (1.03 to 1.47)^b^	1.95 (0.27 to 14.55)	4.50 (0.77 to 8.24)^b^	1.06 (0.88 to 1.27)	1.03 (0.32 to 3.30)	0.61 (−3.03 to 4.24)
Ischemic heart disease	1.16 (1.01 to 1.34)^b^	1.15 (0.91 to 1.44)	21.44 (−7.79 to 50.68)	1.05 (0.91 to 1.22)	0.99 (0.79 to 1.25)	−1.51 (−29.33 to 26.31)
Inflammatory heart diseases	NE^c^	NE^c^	NE^c^	NE^c^	NE^c^	NE^c^
Other heart diseases	1.69 (0.69 to 4.15)	15.18 (0.01 to 23301.99)	0.14 (−0.27 to 0.56)	0.66 (0.21 to 2.09)	0.68 (0.01 to 81.37)	−0.15 (−0.56 to 0.27)
Thrombotic disorders	1.21 (0.80 to 1.83)	1.22 (0.48 to 3.13)	0.28 (−0.72 to 1.28)	0.86 (0.54 to 1.37)	0.65 (0.05 to 7.74)	−0.43 (−1.37 to 0.52)
Neurological						
Cerebrovascular disease	1.51 (0.97 to 2.34)	1.22 (0.47 to 3.19)	1.66 (−4.19 to 7.50)	1.03 (0.59 to 1.79)	0.92 (0.32 to 2.62)	−0.58 (−5.50 to 4.34)
Movement disorders	1.21 (0.83 to 1.77)	1.07 (0.47 to 2.42)	1.58 (−10.34 to 13.50)	0.81 (0.53 to 1.22)	0.98 (0.46 to 2.08)	−0.49 (−13.24 to 12.26)
Cognitive disorders	1.53 (1.22 to 1.92)^b^	1.65 (1.02 to 2.65)^b^	33.03 (14.65 to 51.41)^b^	1.14 (0.89 to 1.46)	1.30 (0.78 to 2.18)	14.35 (−4.04 to 32.73)
Episodic disorders	1.31 (0.93 to 1.82)	1.22 (0.69 to 2.15)	2.26 (−2.81 to 7.32)	0.95 (0.65 to 1.38)	0.75 (0.42 to 1.33)	−2.81 (−7.49 to 1.86)
Other neurological disorders	1.23 (1.04 to 1.47)^b^	1.25 (0.96 to 1.64)	486.09 (−52.92 to 1025.10)	1.03 (0.86 to 1.24)	1.08 (0.82 to 1.42)	162.04 (−367.10 to 691.18)
Peripheral neuropathies	0.79 (0.35 to 1.77)	0.70 (0.14 to 3.27)	−40.80 (−166.44 to 84.84)	1.04 (0.53 to 2.02)	0.94 (0.27 to 3.24)	−9.78 (−151.88 to 132.32)
Sensory disorders	1.10 (0.79 to 1.54)	1.18 (0.71 to 1.97)	0.14 (−0.25 to 0.53)	1.13 (0.82 to 1.56)	1.06 (0.66 to 1.71)	0.05 (−0.34 to 0.44)
Psychiatric						
Mood disorders	0.59 (0.22 to 1.59)	0.71 (0.14 to 3.49)	−0.20 (−0.79 to 0.39)	1.12 (0.52 to 2.36)	1.51 (0.35 to 6.67)	0.30 (−0.34 to 0.94)
Stress and anxiety disorders	1.23 (0.98 to 1.54)	1.28 (0.83 to 1.98)	3.77 (0.05 to 7.49)^b^	1.17 (0.93 to 1.46)	1.31 (0.84 to 2.03)	4.40 (0.53 to 8.27)^b^
Psychotic disorders	0.98 (0.54 to 1.79)	1.59 (0.51 to 5.05)	0.65 (−0.25 to 1.56)	1.08 (0.63 to 1.83)	1.21 (0.44 to 3.36)	0.29 (−0.64 to 1.22)
Endocrine						
Diabetes	1.09 (0.89 to 1.33)	0.98 (0.67 to 1.43)	−0.43 (−3.41 to 2.55)	1.00 (0.80 to 1.24)	0.93 (0.63 to 1.37)	−0.76 (−3.34 to 1.82)
Dyslipidemia	1.12 (1.06 to 1.18)^b^	1.20 (1.09 to 1.33)^b^	13.14 (9.23 to 17.04)^b^	1.07 (1.02 to 1.13)^b^	1.06 (0.96 to 1.17)	4.35 (0.29 to 8.42)^b^
Gastrointestinal						
Gastritis	1.23 (0.93 to 1.62)	1.20 (0.78 to 1.84)	0.09 (−0.11 to 0.28)	1.06 (0.79 to 1.43)	1.15 (0.73 to 1.81)	0.06 (−0.13 to 0.25)
Irritable bowel syndrome	1.00 (0.69 to 1.43)	0.99 (0.59 to 1.64)	NE^c^	1.37 (1.01 to 1.85)^b^	1.35 (0.85 to 2.17)	NE^c^
Acute pancreatitis	1.28 (0.81 to 2.02)	1.12 (0.46 to 2.73)	0.35 (−1.47 to 2.16)	1.05 (0.66 to 1.68)	1.03 (0.36 to 2.94)	−0.06 (−1.93 to 1.82)
Biliary tract disease	1.21 (0.82 to 1.77)	1.18 (0.64 to 2.17)	0.11 (−0.25 to 0.47)	1.44 (1.03 to 2.01)^b^	1.67 (0.73 to 3.87)	0.47 (0.05 to 0.89)^b^
Noninfectious hepatitis and cirrhosis	2.30 (1.12 to 4.72)^b^	2.77 (0.51 to 16.62)	0.03 (−0.00 to 0.05)	2.66 (1.29 to 5.47)^b^	3.99 (0.73 to 25.60)	0.03 (0.00 to 0.06)^b^

Abbreviations: DALY, disability-adjusted life year; HR, hazard ratio; IRR, incidence rate ratio; NE, not estimable.

^a^ HR greater than 1 denotes higher risk of prespecified sequelae, IRR greater than 1 denotes higher incidence rates of prespecified sequelae, DALYs greater than 0 denotes higher health burdens in dengue-infected individuals vs controls.

^b^ Denotes statistical significance at 95% level.

^c^ Not estimable owing to small number of outcomes in either comparator group, or if DALYs by that cause were not available.

## Discussion

This nationwide cohort study found that dengue infection is followed by sustained multisystem morbidity and mortality extending well beyond the febrile phase. Benchmarking against prior acute dengue DALY estimates suggests dengue’s burden in Singapore has been underestimated by 13% to 37%,^3^ consistent with data from Mexico, where persistent symptoms contributed 13% to 43% additional burden.^2^ Given the high incidence of dengue in endemic settings, this postacute disability represents a substantial population health impact. Although these sequelae are proposed to be mediated by persistent immune dysregulation and inflammation, the underlying pathophysiology remains incompletely understood.^1,4,5,6^

Limitations include restriction to adults, reliance on claims-based diagnoses, ecological assignment of dengue serotypes, and exclusion of asymptomatic infections, which likely bias estimates toward the null. Nonetheless, sensitivity analyses yielded consistent conclusions. These results support comprehensive incorporation of long-term postacute complications into dengue burden assessments and argue for postdischarge surveillance in high-risk groups.

## Notes

Supplement 1. eAppendix 1. Additional information on databases used and cohort construction eFigure. Flowchart of cohort construction eAppendix 2. Additional information on pre-specified sequelae outcomes eAppendix 3. Additional information on statistical methods and sensitivity analyses eReferences

Supplement 2. Data Sharing Statement
