# Supplementary material for: Complications, Deaths, and Disability Burden in the 2 Years Following Dengue Infection
Source: JAMA Netw Open. 2026 Feb 12;9(2):e2559108. doi: 10.1001/jamanetworkopen.2025.59108 (PMC12902890; doi:10.1001/jamanetworkopen.2025.59108)
Supplement: Supplement 1. — eAppendix 1. Additional information on databases used and cohort construction eFigure. Flowchart of cohort construction eAppendix 2. Additional information on pre-specified sequelae outcomes eAppendix 3. Additional information on statistical methods and sensitivity analyses eReferences [file jamanetwopen-e2559108-s001.pdf]

Supplemental Online Content

Chow JY, Tan WZ, Wee LE, et al. Complications, deaths, and disability burden in the 2 years following dengue infection. *JAMA Netw Open*. 2026;9(2):e2559108. doi:10.1001/jamanetworkopen.2025.59108

eAppendix 1. Additional information on databases used and cohort construction
eFigure. Flowchart of cohort construction
eAppendix 2. Additional information on pre-specified sequelae outcomes
eAppendix 3. Additional information on statistical methods and sensitivity analyses
eReferences

This supplemental material has been provided by the authors to give readers additional information about their work.

© 2026 Chow JY et al. *JAMA Network Open*.

eAppendix 1. Additional information on databases used and cohort construction

Singapore is a highly urbanised, tropical city-state with a residential population of 6.03 million comprising diverse ethnic groups. Dengue is endemic in Singapore, with persistent non-zero weekly case counts and sustained co-circulation of at least three DENV serotypes over the past 15 years, and periodic outbreaks continue to be reported every few years in the setting of long-standing national *Aedes* control programmes and high case ascertainment.[1,2] Confirmatory diagnostic testing for dengue, including NS1 antigen detection and IgM enzyme-linked immunoassays, is widely accessible across all healthcare settings in Singapore. Under statutory reporting regulations, all confirmed dengue cases are mandatorily notified to the Ministry of Health (MOH) within 24 hours of laboratory diagnosis.[3] Using linked national dengue surveillance registries and healthcare claims databases, we assembled a retrospective cohort of individuals first infected with laboratory-confirmed dengue between 1st January 2013 to 30th June 2022 **(eFigure)**. Given the overlap between the latter part of the study period and the emergence of COVID-19 in Singapore and in light of extensive literature on post-acute sequelae of SARS-CoV-2 infection,[4] we excluded individuals with confirmed SARS-CoV-2 infection documented in the national COVID-19 registry either within 365 days prior to their dengue index date (T_0) or during the post-infection follow-up window of 30 to 730 days.

Post-acute risks of all-cause mortality, hospitalisation, and incident complications across cardiovascular, neurological, psychiatric, autoimmune, renal, and gastrointestinal systems were examined using Singapore's national healthcare claims data (1st January 2012 to 30th June 2024). Healthcare utilisation records are comprehensively captured due to mandatory participation in Medisave, a government-administered medical savings scheme that permits claims at both public and private healthcare institutions, enabling systematic identification of new clinical events across a range of healthcare settings. The **eFigure** outlines the construction of the study cohort.

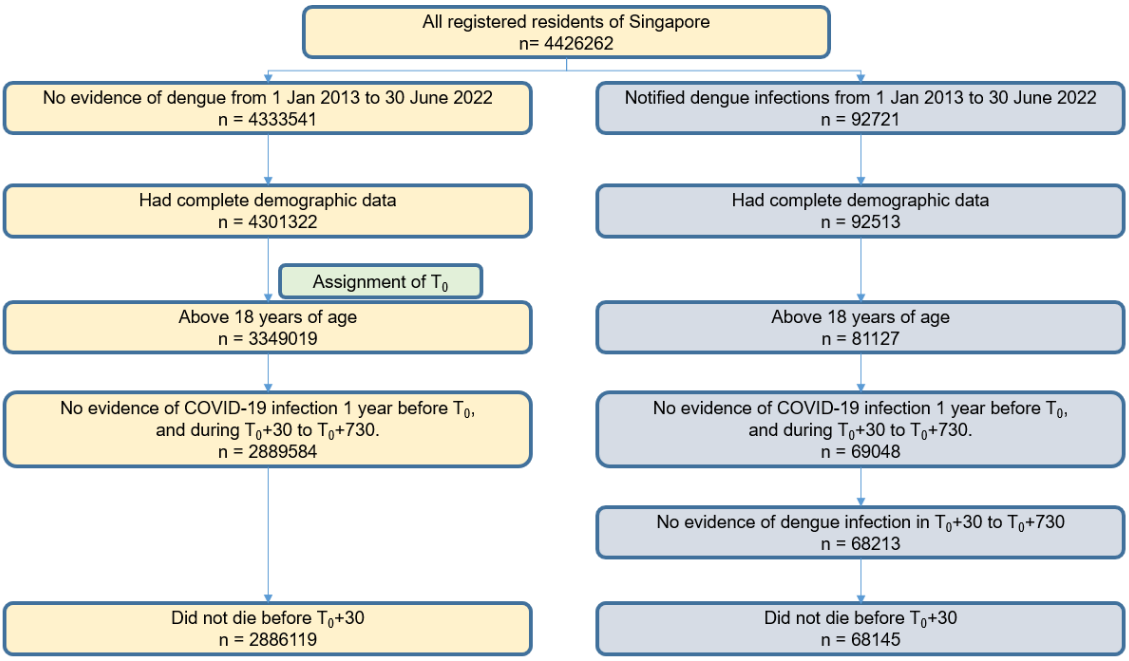

eFigure. Flowchart of cohort construction

© 2026 Chow JY et al. *JAMA Network Open*.

eAppendix 2. Additional information on pre-specified sequelae outcomes

The primary outcomes included all-cause hospitalisation and mortality, alongside new-onset complications involving cardiovascular, neurological, psychiatric, autoimmune, renal, endocrine, and gastrointestinal systems, captured over the post-acute window (T_0 +30 to T_0 +730 days). Diagnoses were based on International-Classification-of-Diseases, Tenth Revision (ICD-10) codes recorded in the national healthcare claims database, based on definitions applied in prior investigations of post-viral sequelae, including those related to dengue and COVID-19.[4,5] Cardiovascular events comprised of dysrhythmias, myocarditis or pericarditis, ischemic heart disease, thrombotic complications, and other cardiac pathologies such as heart failure. Neurological and psychiatric outcomes were grouped together into the neuropsychiatric domain and consisted of cerebrovascular disease, cognitive impairment, extrapyramidal syndromes, episodic disturbances (e.g. seizures, migraine), peripheral neuropathy, musculoskeletal and sensory dysfunction, and a range of psychiatric diagnoses including psychosis, depression, mood instability, and anxiety. Autoimmune conditions were treated as a single composite endpoint due to the rarity of specific diagnoses. Endocrine outcomes were similarly aggregated, except for diabetes, which was examined individually. Renal sequelae were analysed as a single category, as laboratory indices necessary to assess functional impairment (e.g. serum creatinine levels) were not available in the claims dataset. Gastrointestinal endpoints spanned gastritis, irritable bowel syndrome, acute pancreatitis, biliary disease, and hepatitis.

Adjusted covariates included demographic characteristics (age, sex, ethnicity), comorbidity burden as measured by the Charlson Comorbidity Index, prior inpatient admissions within the preceding five years, and socioeconomic status (SES). SES was proxied using housing type, a routinely used indicator in Singapore's national databases.

eAppendix 3. Additional information on statistical methods and sensitivity analyses

New-onset sequelae risk occurring between 30 to 730 days after T_0 were assessed by comparing dengue-infected individuals with individuals with no evidence of dengue infection. For each outcome of interest, a sub-cohort was created by excluding individuals with pre-existing diagnoses recorded within five years prior to T_0. Covariate balance between groups was evaluated using standardised mean differences (SMDs). Covariate imbalance was addressed using overlap weighting,[6] where propensity scores were estimated using logistic regression with dengue exposure status as the dependent variable and all covariates as predictors. Weights were defined as 1 minus the propensity score for exposed individuals and as the propensity score for unexposed individuals. Post-weighting, balance was considered adequate if SMDs were less than 0.1.

To estimate post-acute risks, rates and burdens, (**1**) hazard ratios (HRs) for new-onset complications were derived using competing risks regression with overlap weighting, incorporating death as a competing event. Risk of all-cause mortality was assessed separately using Cox proportional hazards models, with overlap weights employed. (**2**) Incidence rate ratios were estimated using negative binomial regression to estimate the excess cumulative incidence of complications post-dengue infection. Overlap weights were similarly employed here. The incidence rate ratio, defined as the estimated cumulative number of complications in the dengue-infected group, over the population-based control group was reported. (**3**) We computed the excess burdens, defined as the weighted differences in incidence rates between the dengue-infected and population-based control group (**4**) We computed

© 2026 Chow JY et al. *JAMA Network Open*.

the excess disability-adjusted life-years (DALYs) due to dengue, we first obtained the attributable number of complications due to dengue by taking the weighted excess burdens per person day multiplied by the number of dengue person days followed up in our cohort for each complication. This attributable number was then multiplied by the average DALYs per incident case to obtain the excess DALYs due to dengue for that condition. DALYs per incident case were derived from Singapore-specific estimates from cause Level 3 in the Global Burden of Disease (GBD) 2021. Each pre-specified sequelae was mapped to its corresponding Level 3 cause. Subsequently, DALYs per case for individual complications were determined by calculating the proportion of total DALYs attributable to the cause relative to its reported prevalence. For composite outcomes, individual complications were mapped to each composite outcome, and the DALYs per case were computed as the mean of the DALYs across all associated complications.

We obtained estimates of excess and burden risk by the first and second year after exposure to dengue by truncating the follow-up period to either T_0+30 to T_0+365 or T_0+366 to T_0+730 and rerunning our statistical approaches. To examine variations in post-acute outcomes by severity of initial dengue illness, dengue cases were stratified based on whether they were hospitalised during the acute phase. Within each subgroup (non-hospitalised or hospitalised), risks of sequelae were independently estimated relative to population-based controls, applying identical data sources, model specifications, and outcome definitions. Exploratory subgroup analysis by age (18 – 40 years/41–60years/≥61 years), socioeconomic status (1-3, 4-5 room public apartments, private apartments), comorbidity status (CCMI=0, CCMI=1+), ethnicity (Chinese/other ethnicities) and sex (male/female) were conducted to examine potential effect modification.

To assess the robustness of results, several sensitivity analyses were undertaken. First, alternative weighting strategies were applied in the main analysis, including: **(1)** inverse probability weighting (IPW), **(2)** a doubly robust approach combining IPW with covariate adjustment in the outcome regression step, and **(3)** stabilised IPW. Second, negative outcome controls were used to identify possible unmeasured confounding and systematic bias in outcome ascertainment. Here, we explored the risk of new-incident asthma post-dengue infection as the negative outcome control. Asthma risk has been shown to increase following SARS-CoV-2 infection,[7] but not dengue (a non-respiratory virus). This procedure was repeated in the main cohort and all subgroups, applying the same data sources, inclusion criteria, statistical models, and outcome definitions. Third, as our observation period comprised both pre- and post-COVID-19 pandemic periods,[8,9] we assessed if there were major changes in the incidence of studied sequelae over the observation period by computing yearly incidence rates of pre-specified new-onset conditions in the entire adult Singaporean population from January 2013–June 2022. Lastly, we explored the sensitivity of our excess DALY estimates by alternatively taking the attributable number of complications due to dengue as the unweighted excess burdens per person day multiplied by the number of dengue person days followed up in our cohort for each complication. We also computed excess DALYs per incident case were taken as the global average DALYs by cause instead of Singapore-specific DALYs.

All statistical tests reported 95% confidence intervals. Analyses were implemented in R version 4.3.1.

© 2026 Chow JY et al. *JAMA Network Open*.

eReferences

1. National Environment Agency. National Environment Agency, Singapore. Published online January 19, 2023. https://www.nea.gov.sg/dengue-zika/dengue/quarterly-dengue-surveillance-data.

2. Ho SH, Lim JT, Ong J, Hapuarachchi HC, Sim S, Ng LC. Singapore's 5 decades of dengue prevention and control—Implications for global dengue control. *PLOS Neglected Tropical Diseases*. 2023;17(6):e0011400. doi:10.1371/journal.pntd.0011400

3. Wee LE, Lim JT, Tan JYJ, et al. Dengue versus COVID-19: comparing the incidence of cardiovascular, neuropsychiatric and autoimmune complications. *J Travel Med*. Published online July 7, 2024. doi:10.1093/jtm/taae081.

4. Lim JT, Liang En W, Tay AT, et al. Long-term Cardiovascular, Cerebrovascular, and Other Thrombotic Complications in COVID-19 Survivors: A Retrospective Cohort Study. *Clin Infect Dis*. Published online January 25, 2024. doi:10.1093/cid/ciad469.

5. Wee LE, Lim JT, Tay AT, et al. Long-term cardiovascular, cerebrovascular, and thrombotic complications after SARS-CoV-2-Omicron infection: a retrospective cohort study. *Clin Microbiol Infect*. Published online June 20, 2024. doi:10.1016/j.cmi.2024.06.011.

6. Li F, Thomas LE, Li F. Addressing Extreme Propensity Scores via the Overlap Weights. *Am J Epidemiol*. 2019;1;188(1):250-257. doi:10.1093/aje/kwy201.

7. Meng M, Wei R, Wu Y, et al. Long-term risks of respiratory diseases in patients infected with SARS-CoV-2: a longitudinal, population-based cohort study. *EClinicalMedicine*. Published online February 17, 2024. doi:10.1016/j.eclinm.2024.102500.

8. Wee LE, Cherng BPZ, Conceicao EP, et al. Experience of a Tertiary Hospital in Singapore with Management of a Dual Outbreak of COVID-19 and Dengue. *The American Journal of Tropical Medicine and Hygiene*. 2020;103(5):2005-2011. doi:10.4269/ajtmh.20-0703

9. Tang N, Lim JT, Dickens B, et al. Effects of Recent Prior Dengue Infection on Risk and Severity of Subsequent SARS-CoV-2 Infection: A Retrospective Cohort Study. *Open Forum Infect Dis*. Published online July 13, 2024. doi:10.1093/ofid/ofae397.

© 2026 Chow JY et al. *JAMA Network Open*.
