# Supplementary material for: Complications, Deaths, and Disability Burden in the 2 Years Following Dengue Infection
Source: JAMA Netw Open. 2026 Feb 12;9(2):e2559108. doi: 10.1001/jamanetworkopen.2025.59108 (PMC12902890; doi:10.1001/jamanetworkopen.2025.59108)
Supplement: Supplement 2. — Data Sharing Statement [file jamanetwopen-e2559108-s002.pdf]

Data Sharing Statement

Chow. Complications, Deaths, and Disability Burden in the 2 Years Following Dengue Infection. *JAMA Netw Open*. Published February 12, 2026.
doi:10.1001/jamanetworkopen.2025.59108

Data
Data available: No

Additional Information
Explanation for why data not available: The databases with individual-level information used for this study are not publicly available due to personal data protection. Deidentified data can be made available for research, subject to approval by the Ministry of Health of Singapore. All inquiries should be sent to the corresponding author.
